# Preparation of Magnetic Iron Oxide Nanoparticles (MIONs) with Improved Saturation Magnetization Using Multifunctional Polymer Ligand

**DOI:** 10.3390/polym8110392

**Published:** 2016-11-08

**Authors:** Muhammad Irfan Majeed, Jiaojiao Guo, Wei Yan, Bien Tan

**Affiliations:** 1 School of Chemistry and Chemical Engineering, Huazhong University of Science and Technology, Wuhan 430074, China; irfanmajeed2003@gmail.com (M.I.M.); gjj824405637@gmail.com (J.G.); bien.tan@mail.hust.edu.cn (B.T.); 2 Hubei Collaborative Innovation Center for Advanced Organic Chemical Materials, Ministry of Education, Key Laboratory of Green Preparation and Application for Functional Materials, Hubei Key Laboratory of Polymer Materials, School of Materials Science & Engineering, Hubei University, Wuhan 430062, China

**Keywords:** magnetic iron oxide nanoparticles, polymer ligand, biocompatible, multifunctional, saturation magnetization

## Abstract

This paper describes the preparation of ultra-small magnetic iron oxide (Fe_3_O_4_) nanoparticles (MIONs) coated with water-soluble thioether end-functionalized polymer ligand pentaerythritol tetrakis 3-mercaptopropionate-polymethacrylic acid (PTMP-PMAA). The MIONs were prepared by co-precipitation of aqueous iron precursor solution at a high temperature. The polymer modified MIONs were characterized by dynamic light scattering (DLS), transmission electron microscopy (TEM), Fourier transform infrared spectroscopy (FTIR), X-ray powder diffraction (XRD), thermogravimetric analysis (TGA), and vibrating sample magnetometery (VSM). It was found that these MIONs were successfully modified by this water-soluble polymer ligand with a fairly uniform size and narrow size distribution. The dried powder of MIONs could be stored for a long time and re-dispersed well in water without any significant change. Additionally, the polymer concentration showed a significant effect on size and magnetic properties of the MIONs. The saturation magnetization was increased by optimizing the polymer concentration. Furthermore, the 3-(4,5-dimethylthiazol-2-yl)-2-5-diphenyltetrazolium bromide (MTT)-assay demonstrated that these MIONs were highly biocompatible and they could be successfully coupled with fluorescent dye Rhodamine due to the formation of amide bond between carboxylic acid groups of MIONs and amine groups of dye. The obtained results indicated that these multifunctional MIONs with rich surface chemistry exhibit admirable potential in biomedical applications.

## 1. Introduction

Magnetic nanoparticles (NPs) have gained much scientific interest for their unique magnetic properties such as superparamagnetism, high coercivity, low Curie temperature, and high magnetic susceptibility [1,2]. Magnetic iron oxide nanoparticles (MIONs), owing to their advantages such as low toxicity and biocompatibility, are considered to be the most favorable candidates for bio-applications [3] including magnetic resonance imaging (MRI) [4,5], magnetic fluid hyperthermia (MFH) [6,7], magnetic separation and immobilization of biomolecules such as nucleic acids and proteins [8,9], the development of drug delivery systems for controlled release of drugs [10,11], biolabeling and magnetic sensors [12]. Thermal decomposition and co-precipitation are among the most common techniques employed for MIONs preparation but both having advantages and limitations [13]. It is usually desired to develop a simple, one step, cost effective and environment friendly protocol for the preparation of water-soluble, uniform, multifunctional, superparamagnetic iron oxide NPs with a better control over their size, shape and magnetic properties. Furthermore, for bioapplications of MIONs, they should be highly dispersed in aqueous phase and biocompatible having least or no toxicity, which can be achieved through adopting co-precipitation method using multifunctional water-soluble polymers as capping ligands.

Multifunctional water-soluble polymers as capping ligands can play an important role in the preparation of MIONs and other inorganic NPs [14,15,16,17]. In aqueous co-precipitation process, polymer ligands can efficiently control the size and shape of the NPs due to the presence of abundant functional groups such as –COOH, –OH, –NH_2_, etc. These multifunctional polymers render magnetic NPs more stable, water-soluble and uniform in addition to providing them rich surface chemistry which opens up ways for easier post-synthesis modification and functionalization for bio-applications [5,18,19,20,21,22]. Numerous studies have been reported for the synthesis of MIONs using polymer ligands in order to render them water-soluble and biocompatible. However, in most of the cases, the NPs are first prepared through thermal decomposition and then made water-soluble through post-synthesis modification processes such as ligand exchange using water-soluble polymer ligands [23,24].

In this study, we have described the synthesis of MIONs with a multifunctional water-soluble polymer ligand pentaerythritol tetrakis 3-mercaptopropionate-polymethacrylic acid (PTMP-PMAA), following our previous work [20]. Previously, it was demonstrated that PTMP-PMAA, having abundance of carboxylic acid groups, can be successfully used for the stabilization of the MIONs in the co-precipitation procedure. These carboxylic acid groups have ability to cap the growing MIONs in the reaction mixture through coordinating with iron oxide surface and thus stabilize them and control their size and size distribution depending upon the concentration of the polymer ligand. Therefore, the concentration of the polymer ligand or the molar ratio between carboxylic acid groups of the PTMP-PMAA and iron precursors play an important role in preparation of uniform MIONs through aqueous co-precipitation procedure. However, in that report, MIONs prepared with 0.768 mM concentration of PTMP-PMAA had very small size (4.5 ± 0.4 nm) and lower saturation magnetization (45 emu·g^−1^). Those MIONs, due to their high dispersibility and ultra-small size, were successfully used as dual MRI contrast agents [5]. However, due to their lower saturation magnetization, they could not be manipulated in dispersed state with the use of an external magnet, which limits their scope of applications.

Therefore, herein, we report the preparation of MIONs using PTMP-PMAA with improved saturation magnetization through our original high temperature single step co-precipitation method [20] but with few modifications in order to improve the magnetic properties of MIONs, such as lower polymer concentrations as compared to previous report, iron precursors were dissolved in concentrated hydrochloric acid (HCl) instead of 1 M HCl solution in order to prevent their hydrolysis and condensation before the addition of precipitating agents and the inert conditions were maintained, by nitrogen gas bubbling throughout the course of reaction, which not only protects MIONs from critical oxidation but also keeps their size smaller [25].

MIONs prepared using PTMP-PMAA had several carboxylic acid (–COOH) functional groups which provide them excellent dispersibility and stability in aqueous solutions. These MIONs showed high resistance against aggregation in aqueous media over a wide range of pH and salt concentration due to excellent electrostatic and steric stabilization provided by the polymer ligand. These MIONs can be dried by evaporating solvent and stored as powder for several months without any undesired changes in their chemical and physical properties. The polymer ligand offers MIONs better chemical stability against oxidation which otherwise leads to a decrease in their magnetic properties. Furthermore, cytotoxicity analysis of the MIONs proved them to be biocompatible even at their high concentration up to 500 µg·mL^−1^. Bio-applicability of these NPs was demonstrated by successfully conjugation of MIONs with a fluorescent dye Rhodamine (Rh 110). Such bimodal detection systems based on fluorescent molecules and magnetic NPs can facilitate the deep tissue imaging by combined optical and MRI techniques [26]. The fabrication of novel targeted luminescent and magnetic NPs with multifunctional water-soluble polymers would play a vital role in the development of contrast agents for diagnosis, imaging and therapeutic technologies of the new era [26,27]. Finally, it was concluded that MIONs stabilized with PTMP-PMAA were extremely biocompatible and can be used for several bio-applications due to their chemically rich surface providing numerous opportunities for their conjugation with a variety of therapeutic, targeting, and labeling agents.

## 2. Materials and Methods

### 2.1. Materials

All chemicals were of analytical grade and were used as received without any further purification, unless otherwise described. Methacrylic acid (MAA, 99%), 2,2′-azobisisobutyronitrile (AIBN, 98%), anhydrous ethanol, anhydrous acetone and anhydrous diethyl ether were purchased from National Medicines Corporation Ltd. of China (Beijing, China). Pentaerythritol tetrakis 3-mercaptopropionate (PTMP, 99%) was obtained from Aldrich (St. Louis, MO, USA). Ferric Chloride (FeCl_3_·6H_2_O, 99%), ferrous sulfate (FeSO_4_·7H_2_O, 99%), hydrochloric acid (HCl, 38%) and ammonium hydroxide (NH_4_OH, 28%) were obtained from Sinopharm Chemical Reagent Co. Ltd. (Shanghai, China) *N*-(3-dimethylamino-propyl)-*N*′-ethylcarbodiimide hydrochloride (EDC) and *N*-hydroxysuccinimde (NHS) were purchased from Aladdin Reagent Co., Ltd. (Shanghai, China). Fetal bovine serum (FBS) and Dulbecco’s modified eagle’s medium (DMEM) were obtained from Gibco (Basel, Switzerland). The 3-(4,5-Dime-ltetrazolium bromide) (MTT) cell proliferation assay kit was received from Amresco (Solon, OH, USA). Milli-Q water was used in the preparation and subsequent application experiments.

### 2.2. Synthesis and Characterization of Polymer Ligand PTMP-PMAA

**Scheme 1:**
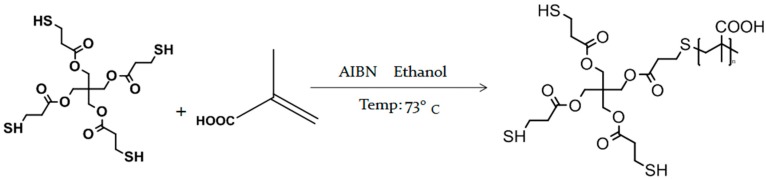
Synthesis of polymer ligand pentaerythritol tetrakis 3-mercaptopropionate-polymethacrylic acid (PTMP-PMAA).

PTMP-PMAA was synthesized by free radical polymerization of monomer methacrylic acid (MAA) using pentaerythritol tetrakis 3-mercaptopropionate (PTMP) as chain transfer agent as described in previous reports [28,29,30]. The molar ratio of monomer to chain transfer agent helped to control the molecular weight of the polymer. Scheme 1 represents the polymer synthesis process.

In a typical preparation for PTMP-PMAA, methacrylic acid (MAA, 5 g, 58 mmol), pentaerythritol tetrakis 3-mercaptopropionate (PTMP, 0.56 g, 1.16 mmol, 2% of monomer) and 2,2′-azobisisobutyronitrile (AIBN, 0.095 g, 0.58 mmol, 1% of monomer) were added to EtOH (25 mL) in a three-necked round-bottomed flask, equipped with a reflux condenser and mechanical stirrer. The temperature of the reaction mixture was maintained at 75 °C for 5 h under Nitrogen with vigorous stirring. At the end of this period, the reaction mixture was left to cool down to room temperature and then the products were isolated by precipitation into cold diethyl ether. The polymer was collected by filtration on a Buchner funnel, and the solvent and monomer residues were removed by evaporation to constant mass using a vacuum oven set at 45 °C. A fraction of low molar mass polymer, un-reacted monomer and some oligomers remaining after reaction are removed during the precipitation step. The yield obtained was 80% for this reaction. ^1^H-NMR spectra were recorded on a 400 MHz Bruker AV400 spectrometer (Billerica, MA, USA) using *d*_6_-DMSO as a solvent in a 5 mm quartz NMR tube at room temperature using the δ scale and were consistent to the previous reports [28,29]. The molecular weights of polymer were determined by gel permeation chromatography (GPC) on Agilent 1100 instrument (Santa Clara, CA, USA) using THF as mobile phase after its methylation with TMS-diazomethane.

### 2.3. Synthesis of MIONs

**Scheme 2:**
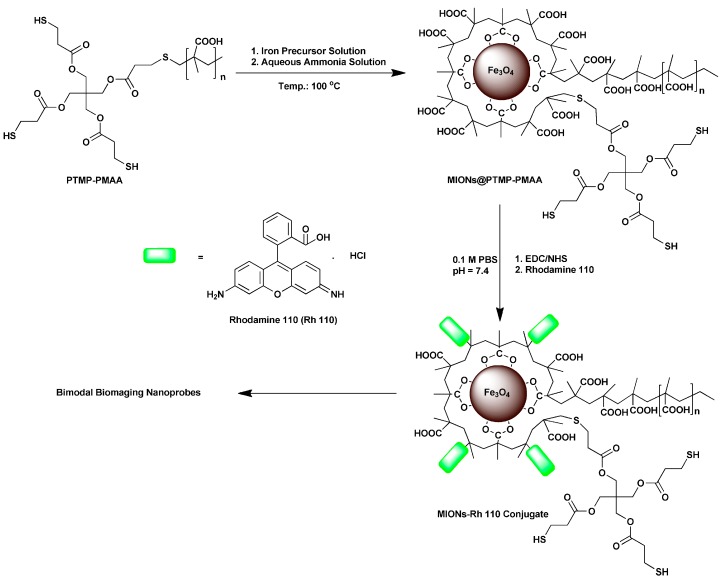
Graphical representation of the synthesis of PTMP-PMAA functionalized MIONs and their subsequent functionalization with Rhodamine 110.

MIONs synthesis process was adopted from our previous work with few modifications [20,25]. Typical procedure involves the co-precipitation of aqueous iron precursor solution with ammonia in the presence of polymer ligand at high temperature (Scheme 2). Brief experimental details of the MIONs synthesis using polymer ligand PTMP-PMAA are provided in the following paragraph.

Briefly, in a 500 mL four-necked round-bottom flask equipped with reflux condenser, thermometer and nitrogen supply, 300 mL of Milli-Q water was added. The water was purged with nitrogen gas to remove oxygen and was heated to reflux in oil bath with magnetic stirring. When temperature reached 80 °C, the polymer ligand (PTMP-PMAA) was introduced to flask to make the aqueous solution of polymer ligand (0.072 mM, pH = 4). When the temperature reached at 100 °C, the iron precursor solution comprising of FeCl_3_·6H_2_O (3.24 mmol) and FeSO_4_·6H_2_O (1.62 mmol) in 6 mL of concentration HCl was added and the 90 mL of concentration NH_4_OH was added within 5 s. Upon addition of iron precursor solution, the color of the reaction mixture became yellow and upon addition of ammonia solution the color of the reaction mixture turned to dark black suddenly indicating the formation of iron oxide NPs. The temperature of the reaction mixture dropped to 85 °C upon the addition of iron precursor and ammonia addition and it took ~15 min to raise the temperature to 100 °C again and then the reaction was allowed to continue for 2 h at this temperature with vigorous magnetic stirring and constant nitrogen bubbling. After 2 h the heating was stopped and reaction mixture allowed cooling down to room temperature under nitrogen and then solvent was removed by rotary evaporator and solution concentrated to 60 mL and dialyzed against Milli-Q water for 72 h using dialysis membrane with molecular weight cut-off value 14,000 kDa. The dried NPs powder was obtained by evaporation of dialyzed NPs black suspension using rotary evaporator, washing with acetone and then drying in vacuum oven to a constant weight. The yield obtained was ~90%. Different samples of MIONs were prepared in the same way but with different concentrations of polymer ligand PTMP-PMAA, in order to obtain MIONs with optimum size and magnetic properties.

### 2.4. Characterization of MIONs

The particle size of the MIONs was determined by a high performance particle sizer (Brookehaven Nano DLS 90Plus/BI-MAS, Holtsville, NY, USA) with multi angle particle sizing option (from Brookhaven Instruments Co., Holtsville, NY, USA) with an effective detection capability of 0.6 to 6000 nm. The zeta potential values were determined by a Brookhaven ZetaPlus (Brookhaven Instruments Co., Holtsville, NY, USA) at 25 °C using the folded capillary cells. Data were obtained using a monomodal acquisition and was fit according to the Smoluchowski theory. After filtration with aqueous membrane (Φ = 13 mm, 0.22 μm), the samples were analyzed using ultrapure water as solvent (pH = 7). These measurements were run at least three times with independent particle batches. Transmission electron microscopy images were recorded on a JEOL-2100 electron microscope (Akishima, Tokyo, Japan) operating at an acceleration voltage of 200 kV. TEM samples were prepared by the slow evaporation of a drop of dilute aqueous solution of MIONs onto carbon-coated copper grids (400 mesh). Images were recorded with a Gatan 794 CCD camera (Pleasanton, CA, USA). Size distribution graphs were prepared by analyzing about 200 individual NPs on each TEM image by ImageJ software. X-ray diffraction (XRD) was recorded on X’Pert PRO XRD spectrometer (PANalytical B.V., Kassel, Holand) using Cu Kα (λ = 1.54056 Å) radiation in the 2θ range of 10°–80°.

The UV–vis spectra were recorded using Lambda 35 UV–vis spectrophotometer (Perkin-Elmer, Waltham, MA, USA). The infrared spectra were recorded by a Fourier transform infrared (FTIR) spectrometer (Bruker Vertex 70, Billerica, MA, USA) equipped with an attenuated total reflection (ATR) accessory. Raman spectra were measured on a HR JOBIN YVON spectrometer using a laser of 632 nm and a 25% filter. Thermogravimetric analysis (TGA) measurements were made using TGA Q500 (TA Instruments, New Castle, DE, USA) at a heating rate of 10 °C·min^−1^ from room temperature to 900 °C in oxygen free atmosphere. The saturation magnetizations (*M*s) of the MIONs were measured at 26.8 °C on a Lakeshore 7400 Series vibrating sample magnetometer (Lake Shore Cryotonics, Westerville, OH, USA). All the magnetization data were normalized to the same weight.

### 2.5. Cytotoxicity Analysis of MIONs

Toxicity analysis of the MIONs was carried out by MTT-assay using HepG2 cells. HepG2 cells were cultured in Dulbecco’s Modified Eagle Medium (DMEM) supplemented with 10% fetal bovine serum (FBS), 100 µg·mL^−1^ streptomycin and 100 U·mL^−1^ penicillin, in a humidified incubator at 37 °C with a 5% CO_2_ atmosphere. Cell viability was determined by MTT-assay. HepG2 cells were seeded into a 96-well plate with a cell density of 1 × 10^4^ cells per well and suspended in DMEM supplemented with 10% FBS and incubated for 24 h at 37 °C in a 5% CO_2_ atmosphere. After that, the cell culture medium was replaced with fresh medium containing different concentrations of (25, 50, 100, 200, 500 and 1,000 µg·mL^−1^) MIONs, PTMP-PMAA and MIONs@PTMP-PMAA in triplicate. A control experiment with only cell culture medium without any NPs or polymer was also carried out in each case. Plates were placed at 37 °C in a humidified 5% CO_2_ incubator and MTT-assay was performed after 24, 48 and 72 h.

For MTT-assay, briefly cell culture media were aspirated and 20 μL MTT (5 mg·mL^−1^) [3-(4,5-dimethylthiazol-2-yl)2,5-diphenyltetrazolium bromide] in FBS free DMEM was added to each well and incubated for another night at 37 °C in a humidified 5% CO_2_ incubator. After incubation, MTT solution was removed and 150 μL DMSO was added in each vial to dissolve newly formed formazan crystals. The plates were placed on a swing bed for 10 min and then absorbance was recorded at 490 nm using a micro plate reader (Thermo Electron Corporation, Waltham, MA, USA). The absorbance was recorded in triplicate in each case with subtraction for plate absorbance at 650 nm and percentage cell viability was calculated as the ratio of mean absorbance of triplicate readings with respect to mean absorbance of control wells:

Cell viability = (*I*_sample_/*I*_control_) × 100

### 2.6. Conjugation of MIONs@PTMP-PMAA with Rhodamine 110

MIONs@PTMP-PMAA, EDC/NHS, and Rh 110 were separately dissolved in phosphate buffered saline (PBS) solution (1 mL, pH = 6.8). The concentration of Rh 110 was 0.1 and 1.0 mg·mL^−1^ for the other reagents. EDC (1.45 mL) and NHS (400 µL) were sequentially added to Fe_3_O_4_ solution (10 mL) and then Rh 110 solution (2.8 mL) was added and the mixture was stirred for 24 h at room temperature. The resulting conjugate (MIONs-R 110) was purified by extensive dialysis against distilled water for three nights. As a control, a dye solution with same molar concentration was also dialyzed for three nights.

## 3. Results and Discussion

### 3.1. Synthesis and Characterization of PTMP-PMAA

**Figure 1:**
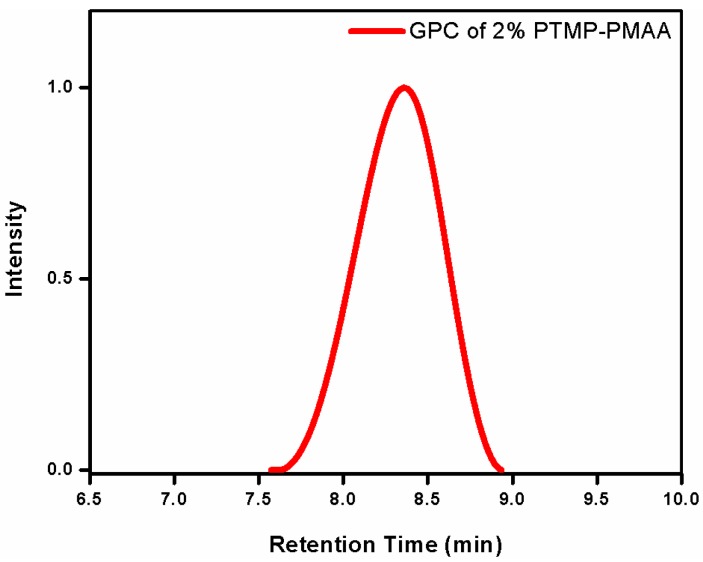
GPC (gel permeation chromatography) curve of the PTMP-PMAA obtained with 2% pentaerythritol tetrakis 3-mercaptopropionate (PTMP).

Multi-functional water-soluble polymer ligand PTMP-PMAA was synthesized using pentaerythritol tetrakis 3-mercaptopropionate as chain transfer agent by free radical polymerization of monomer methacrylic acid (MAA) as described previously (Scheme 1). GPC elution curve of polymer is shown in Figure 1 and its molecular weights are given in Table 1.

^1^H-NMR spectra were recorded on a 400 MHz Bruker AV400 spectrometer using *d*_6_-DMSO as a solvent in a 5 mm quartz NMR tube at room temperature using the δ scale. The ^1^H-NMR spectra were consistent to the previous works [28,29]. The molecular weights of the polymer ligands were calculated based on the ratio of monomer units attached to the terminal group in the ^1^H-NMR spectra and compared with molecular weights determined by GPC (data not given). Chemical structure of the polymer was confirmed by ^1^H-NMR spectroscopy and data as given were consistent with our previous work [28,29].

PTMP-PMAA (*d*_6_-DMSO) δ (ppm): δ0.81~1.14; δ1.42~1.54; δ1.61~1.28; δ2.24~2.40; δ2.51~2.84; δ3.17~3.50; δ3.91~4.40.

The as synthesized polymer was soluble in EtOH, MeOH, H_2_O and DMSO. In order to determine the molecular weight of polymer ligand by GPC it was transferred to the THF by converting it into methyl ester using TMS-Diazomethane reagent according to the previous work [30,31]. GPC was performed with an Agilent 1100 instrument using refractive index detector (RID) and THF was used as eluent at a flow rate of 1.0 mL/min at 23 °C. The calculated molecular weights were based on a calibration curve for polystyrene standards of narrow polydispersity (Polymer Laboratories). As expected, the molecular weight of the polymer was decreased with the increase in concentration of chain transfer agent.

### 3.2. Synthesis and Characterization of Polymer Stabilized MIONs

MIONs were synthesized by co-precipitation of aqueous iron precursor solution containing Fe^3+^ and Fe^2+^ (molar ratio 2:1) by ammonia in the presence of PTMP-PMAA. The size, shape and magnetic properties of the MIONs were controlled by using different molecular weight and concentration of polymer ligand, during their preparation. This polymer ligand has already been proven to be a good capping ligand for the synthesis of cobalt and gold NPs by some of us [21,28,29].

It is known that rapid injection of precursors results in super saturation of chemical species in the reaction mixture which leads to an initial burst of nucleation at once followed by the growth of nuclei leading to the formation of monodisperse inorganic NPs, whereas slow drop-wise addition of precursors results in continuous nucleation and growth process in parallel resulting in broad size distribution of NPs [32]. However, in the case of magnetite NPs, the process is more complicated and it is difficult to control the nucleation and growth processes because iron precursors are initially hydrolyzed in alkaline conditions and then are condensed into iron oxide. Therefore, we dissolved iron precursors in concentrated HCl (38%) in order to avoid their hydrolysis and condensation prior to the addition of precipitating agents. Both iron precursor’s solution and ammonia were rapidly added into the boiling aqueous solution of polymer under nitrogen atmosphere with vigorous stirring in order to keep nucleation and growth processes separate. Rapid injection of precursor is widely used for uniform inorganic NPs synthesis in organic phase by thermal decomposition method [32,33]. The preparation of ultra-small and uniform magnetic NPs with high crystallinity and magnetization through usual co-precipitation method at room temperature is a tedious job, therefore we have carried out co-precipitation process at high temperature (100 °C) [20], which increases the reaction rate due to increased diffusion of active species resulting in the formation of NPs with a narrow size distribution [20].

Polymer ligand (PTMP-PMAA) molecules cap the newly formed NPs through interaction between carboxylic acids groups and the iron atoms on NPs surface and thus restrict their further growth by compensating the surface energy due to electrostatic and steric stabilization which yields uniform as well as highly stable NPs. We have already proved this hypothesis by taking samples at different time intervals showing little further growth of NPs with the passage of time [20]. It was also noticed that the use of different ferrous precursor (FeCl_2_·4H_2_O instead of FeSO_4_·7H_2_O) had no effect on shape, size and size distribution of the MIONs. Moreover, in comparison to the thermal decomposition method, this method involves no use of expensive and toxic organic precursors or solvents but reduces the reaction time and temperature; therefore, it is more economical and eco-friendly.

#### 3.2.1. Effect of Polymer Concentration on Size of MIONs

As described earlier the purpose of this research work was to improve the magnetic properties of our previous reported MIONs prepared with PTMP-PMAA [20]. In that report, MIONs prepared with 0.768 mM concentration of PTMP-PMAA had hydrodynamic diameter <10 nm determined by DLS and core size of 4.5 ± 0.4 nm as determined by TEM. Those MIONs were highly water soluble but their small size and lower magnetization (45 emu·g^−1^) restricted their applications such as magnetic separation. Therefore, herein, we gradually decreased the concentration of PTMP-PMAA from 0.768 mM all the way down to zero during preparation of MIONs and optimized the polymer concentration to obtain MIONs with higher saturation magnetization without compromising their size, dispersibility and stability.

**Figure 2:**
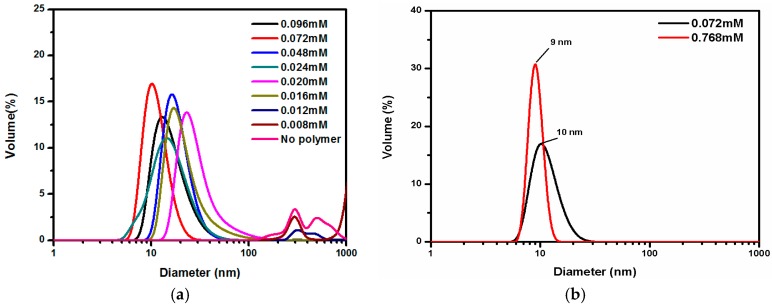
(**a**) Dynamic light scattering (DLS) curves of the MIONs prepared with different concentrations of PTMP-PMAA all lower than 0.768 mM; and (**b**) DLS curves of the MIONs prepared with 0.768 and 0.072 mM concentration of PTMP-PMAA.

Figure 2a shows the DLS curves of the MIONs prepared with different PTMP-PMAA concentrations and it was noticed that the hydrodynamic diameters of the MIONs prepared with polymer concentrations as low as 0.072 mM did not result in the increase in the size of the MIONs. However, when the polymer concentration was decreased beyond 0.072 mM, the size of the MIONs noticeably increased. Figure 2b represent the DLS curves of the MIONs prepared with 0.072 and 0.768 mM PTMP-PMAA representing their hydrodynamic diameters to be 9 and 10 nm respectively, which strongly indicates that the a ten times decrease in polymer concentration did not much affect the size of the MIONs. Therefore, 0.072 mM was considered to be the lowest effective polymer ligand concentration and thus the MIONs prepared with 0.072 mM PTMP-PMAA were selected for further characterization through TEM analysis and the results were compared with those of 0.768 mM PTMP-PMAA.

**Figure 3:**
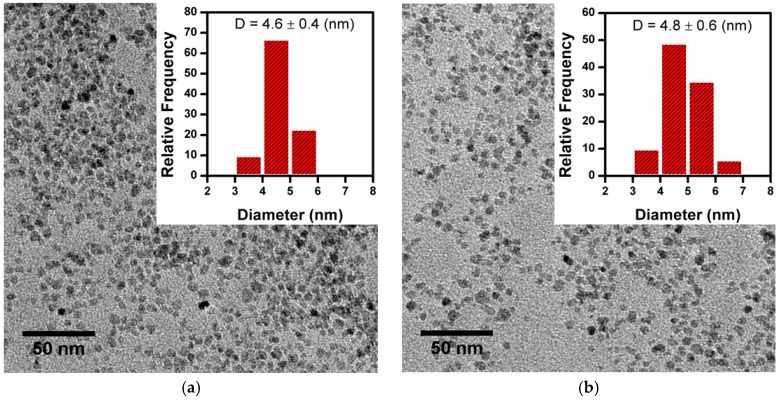
TEM image and histogram (insert) of MIONs prepared with: (**a**) 0.768 mM PTMP-PMAA; and (**b**) 0.072 mM PTMP-PMAA.

In order to determine the actual iron oxide core size of these MIONs, we performed the TEM analysis of the MIONs prepared with 0.072 and 0.768 mM concentrations of PTMP-PMAA. It was interesting to note that the actual size of the MIONs was almost half of their hydrodynamic diameters suggesting the presence of extended polymer shell around these nanoparticles when dispersed in aqueous solutions. The TEM size of the MIONs prepared with 0.768 mM PTMP-PMAA was found to be 4.6 nm with a narrow size distribution of 0.4 nm (Figure 3a) and these results were in complete agreement with the previous report [20]. However, the TEM analysis of the MIONs prepared with 0.072 mM PTMP-PMAA revealed that their size was 4.8 nm with a size distribution of 0.6 nm (Figure 3b), which is very much comparable to the MIONs prepared with 0.768 mM PTMP-PMAA. The slight increase in size and size distribution of these MIONs in comparison to the ten times decrease in the polymer concentration can be considered as insignificant and legitimate.

**Figure 4:**
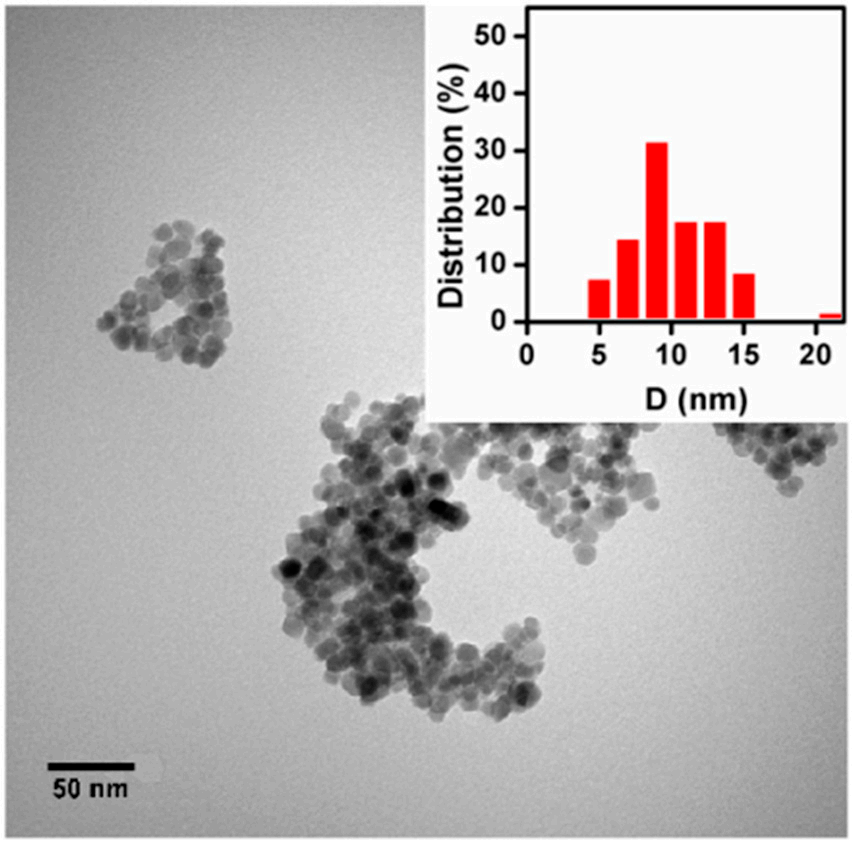
TEM image of Bare MIONs with histogram (inset).

Conversely, the MIONs obtained without PTMP-PMAA had TEM diameter more than 10 nm with very large size distribution range 2.9 nm (Figure 4) and were not very well dispersed in aqueous solution as they tended agglomerate and settle down under force of gravity within a few minutes, perhaps due to their large size, high surface energy, etc. These results revealed that the concentration of the PTMP-PMAA (carbonyl groups to iron precursor ratio) played an important role, and is a critical factor in determining size and size-distribution of the MIONs. It should be noted that the DLS diameter of the bare MIONs was observed to be more than 70 nm because of their high agglomeration, which was further confirmed by TEM (Figure 4).

In conclusion, it was found that the MIONs were successfully synthesized with small size (4.8 ± 0.6 nm) using 0.072 mM PTMP-PMAA which is almost ten times lower concentration as compared to 0.768 mM used previously without significant increase in the size and size distribution of the MIONs. Furthermore, it would also improve the magnetic properties of the MIONs by lowering the polymer contents percentage.

#### 3.2.2. Characterization of MIONs

Further characterization of the MIONs was performed by taking FTIR, TGA, XRD and VSM analysis of the MIONs prepared with 0.072 and 0.768 mM PTMP-PMAA, and results were compared.

**Figure 5:**
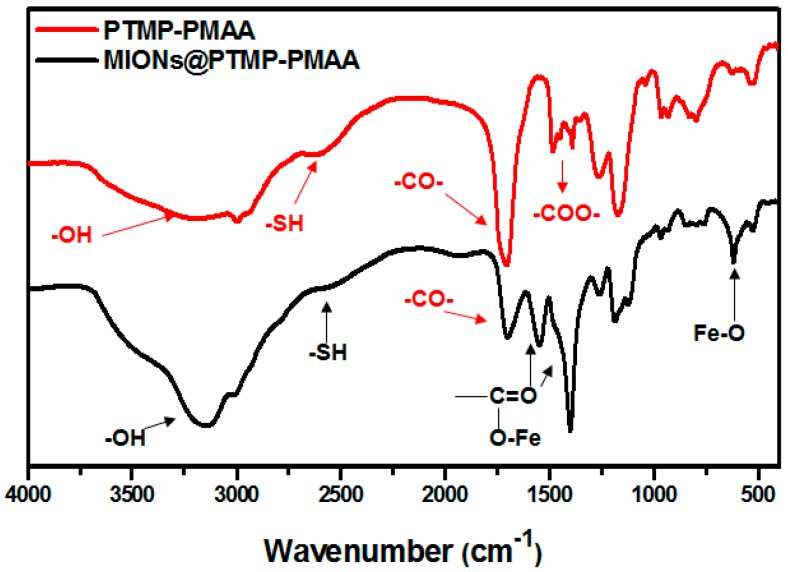
Fourier transform infrared spectroscopy (FTIR) spectra of PTMP-PMAA and MIONs@PTMP-PMAA.

In order to know the surface chemistry of MIONs, they were characterized by Fourier transform infrared spectroscopy (Figure 5). In FTIR spectrum of polymer ligand, a strong absorption peak at 1704 cm^−1^ corresponds to the asymmetric stretching of carbonyl (–CO–) group of polymer ligand, which is extensively reduced in spectrum of MIONs with polymer indicating the attachment to the surface of iron oxide NPs [34]. Additionally, a broad peak at 3124 cm^−1^ is attributed to the O–H stretching of carboxylic acid groups and is observed in FTIR spectra of PTMP-PMAA and MIONs@PTMP-PMAA [28,35]. Furthermore, the asymmetric and symmetric stretching bands of carboxylate (–COO–) present in pure polymer ligand spectrum are shifted from 1485, 1390 to 1556 and 1402 cm^−1^, respectively, in the spectrum of MIONs protected with polymer ligand, which confirms the presence of polymer ligand on NPs surface. Finally, the characteristic peaks appearing at 592 and 448 cm^−1^ are assigned to the torsion vibration and stretching mode of Fe–O bond of magnetite present only in FTIR spectra of MIONs with PTMP-PMAA [35,36].

**Figure 6:**
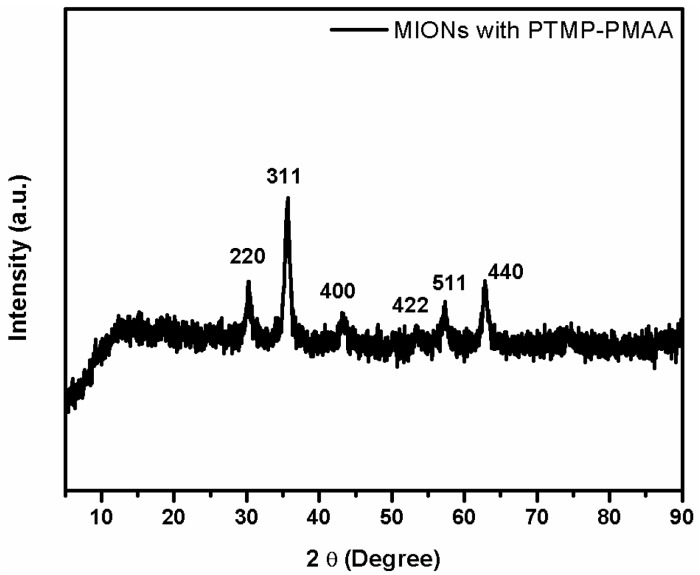
X-ray powder diffraction (XRD) spectrum of MIONs prepared with PTMP-PMAA.

The crystalline structure of the MIONs was determined by XRD measurement as shown in Figure 6. The peaks present at 2θ = 30.1°, 35.5°, 43.3°, 53.4°, 57.3° and 62.7° corresponding to (220), (311), (400), (422), (511) and (440) reflections of magnetite, respectively, can be clearly seen, indicating the presence of crystalline spinel structured magnetite (Fe_3_O_4_) phase of iron oxide [37]. However, the presence of Fe_2_O_3_ is not completely ruled out as the phase of the iron oxide in magnetic NPs cannot be confirmed from simple FTIR and XRD as they are less sensitive to them [20].

**Figure 7:**
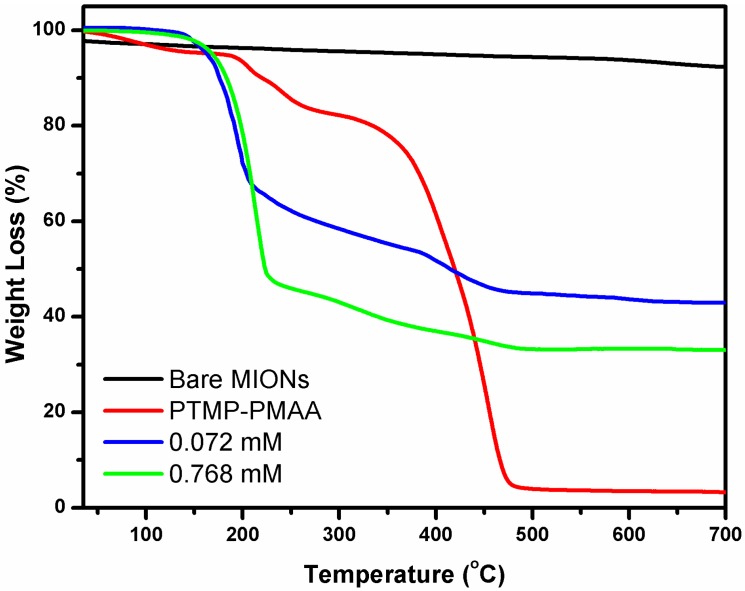
Thermogravimetric analysis (TGA) curves of PTMP-PMAA, MIONs with PTMP-PMAA (0.072 mM and 0.768 mM) and bare MIONs.

To further investigate the thermal stability of the MIONs, and to deduce the iron oxide and polymer contents of the NPs, thermal gravimetric analysis was performed for PTMP-PMAA, MIONs prepared with 0.072 and 0.768 mM PTMP-PMAA as well as for bare MIONs prepare without the use of any polymer ligand (Figure 7). It can be seen that neat polymer ligand (PTMP-PMAA) was almost completely decomposed at 480 °C with almost no residue left. The magnetic iron oxide and polymer contents of the MIONs prepared with 0.768 mM PTMP-PMAA were approximately 33% and 67%, respectively. However, the iron oxide and polymer contents of the MIONs prepared with 0.072 mM PTMP-PMAA were 42% and 58%, respectively, as expected. Therefore, from TGA analysis, it was concluded that the magnetic iron oxide wt % in the MIONs with PTMP-PMAA (0.072 mM) was higher than those prepared with 0.768 mM PTMP-PMAA which contributed towards the higher saturation magnetization of the MIONs in this case.

**Figure 8:**
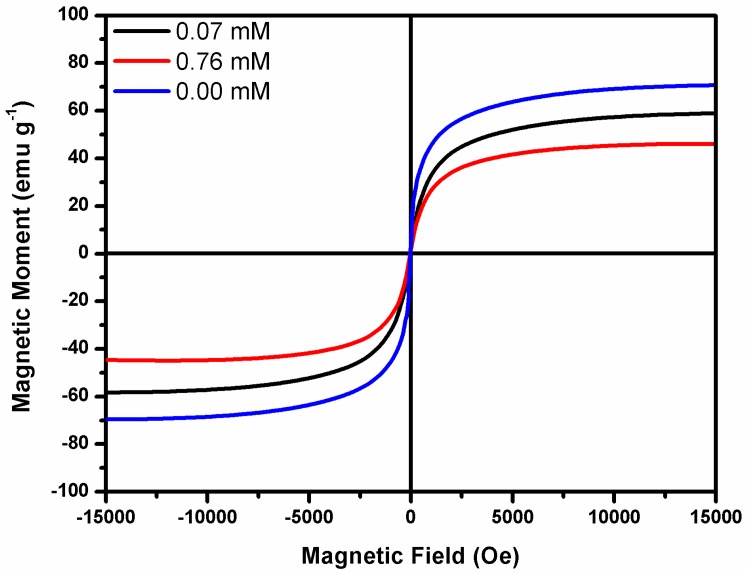
Magnetization curves of MIONs prepared with 0.072, 0.768 and 0.00 mM concentration of PTMP-PMAA.

Saturation magnetization of MIONs prepared with 0.072 and 0.768 mM PTMP-PMAA ligands was determined by Vibrating Sample Magnetometer (VSM) at room temperature. In order to calculate the exact magnetization of these MIONs, the effect of magnetically dead polymer layer was excluded and then compared with magnetization of bare MIONs without polymer (Figure 8). The corrected saturation magnetization of the MIONs prepared with 0.768 mM PTMP-PMAA, having TEM diameter about 4.6 nm, was found to be 45 emu·g^−1^, which is comparable to the literature for MIONs of comparable dimensions [20]. The low saturation magnetization of 4.6 nm MIONs compared to the bare MIONs is due to their ultra-small size as saturation magnetization of magnetic NPs is directly related to their size [38]. However, it was interesting to note that the saturation magnetization of our MIONs prepared with 0.072 mM PTMP-PMAA with TEM diameter 4.8 nm was found to be 58 emu·g^−1^, which is much higher than that of MIONs prepared with 0.768 mM. We attribute this increase in the saturation magnetization of the MIONs to the decrease in magnetically dead polymer contents of the MIONs as mentioned earlier.

However, the magnetization of the MIONs was still much lower than that of bare MIONs prepared without polymer ligand (Figure 8). Therefore, it can be concluded that water-soluble MIONs with comparable size were prepared with ten times less polymer concentration as compared to previous report [20], which resulted in a great increase in the magnetization of the MIONs.

**Figure 9:**
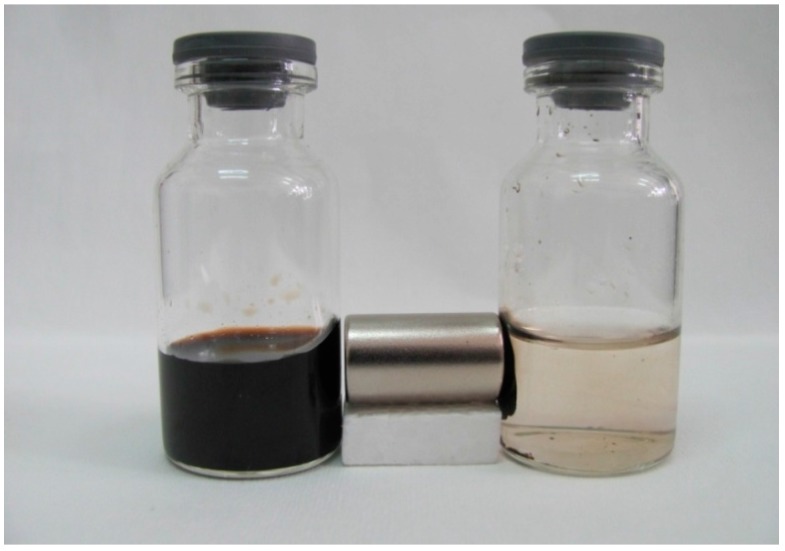
Aqueous dispersion of the MIONs prepared with: 0.768 mM (**left**); and 0.072 mM (**right**) concentrations of PTMP-PMAA under the effect of external magnetic (1.5 T) after 1 h.

It is interesting to note that the increase in the saturation magnetization of the MIONs made them able to be collected with the help of an external magnet from their aqueous solution without compromising their stability and dispersibility, as shown in the Figure 9. We believe that this would increase the scope of applications of these MIONs such as the magnetic separations of biomolecules like proteins, nucleic acids and even cells.

Dried MIONs prepared with PTMP-PMAA were highly stable and readily redispersible in water, pH and salt stability tests were performed with aqueous dispersions of MIONs prepared with PTMP-PMAA (0.072 mM) and were found to be stable within a wide range of pH (5–10) and high concentrations (1 M) of salt (NaCl). High stability of the MIONs dispersions can be attributed to large negative zeta potential (−49 mV) and excellent electrostatic as well as steric stabilization by polymer ligand owing to the large abundance of carboxylic acid groups [25].

#### 3.2.3. Toxicity Analysis of MIONs

**Figure 10:**
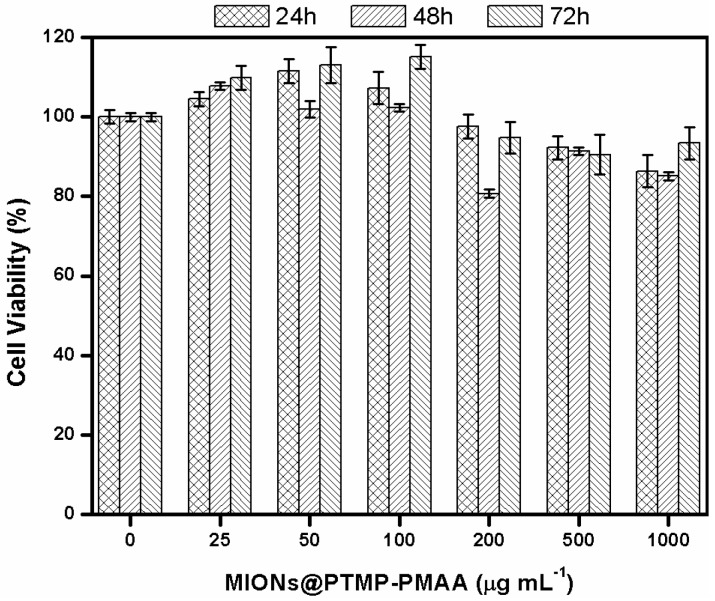
Cell viability of HepG2 determined by MTT-assay after 24, 48 and 72 h of incubation with various concentrations of MIONs@PTMP-PMAA.

Biocompatibility of the MIONs@PTMP-PMAA was determined through their in vitro cytotoxicity analysis performed on HepG2 cells as demonstrated in experimental section. Figure 10 shows the percent cell viability of HepG2 cells, which was determined by MTT-assay, after incubation of cells with different concentrations of MIONs prepared with 0.072 mM PTMP-PMAA for 24, 48 and 72 h. Cell viability determined at zero concentration of MIONs or polymer ligand was taken as 100%. These in vitro cytotoxicity tests clearly demonstrate that MIONs@PTMP-PMAA were completely non-toxic and cells survived well even at higher concentrations (500 µg·mL^−1^) due to nontoxic nature of PTMP-PMAA [5], however, cell viability more than 100% in few cases was maybe due to cell proliferation. In opposite to that, bare MIONs have time and dose dependent high toxicity as described by us previously [25], which was attributed to the known fact that bare MIONs act as a source of ferrous ions and highly reactive hydroxyl radicals causing oxidative and free radical stress to the cells [39].

### 3.3. Functionalization of MIONs with Fluorescent Dye

It was determined through characterization that MIONs@PTMP-PMAA had high water dispersibility and excellent stability in a wide range of pH and salt concentration and multifunctional surface chemistry, due to polymer shell around them, which make them highly suitable for bio-applications such as contrast agents for MRI, magnetic separation of biomolecules and cells, and development of NPs based drug delivery systems. Furthermore, the presence of abundant of carboxylic acid (–COOH) and thiol (–SH) groups on MIONs surface also provide great opportunities to simultaneously modify these NPs with targeting, tracing and therapeutic agents for multipurpose bio-probes. We demonstrated the functionalization of these MIONs with a fluorescent dye molecule, Rhodamine 110 (Rh 110) bearing primary amine group through EDC/NHS coupling reaction, which is the easiest and most feasible reaction of linking molecules through primary amine and carboxylic acid groups by amide bond formation.

**Figure 11:**
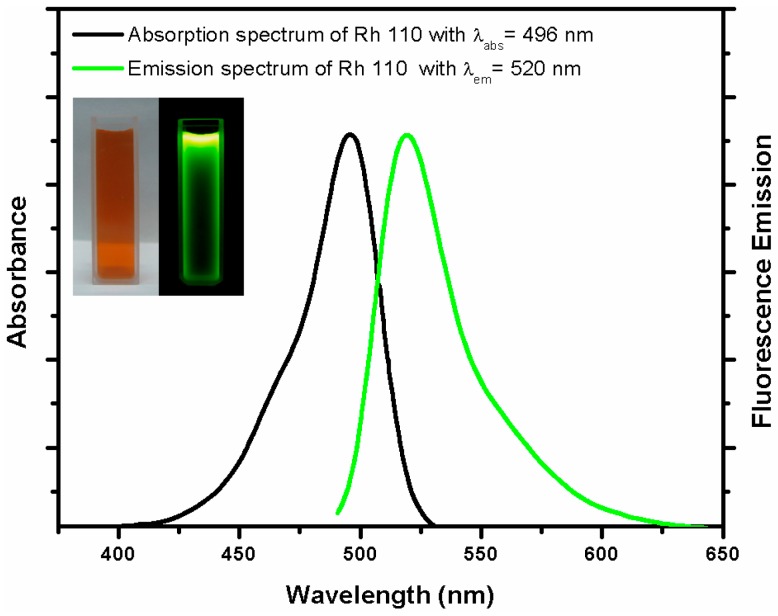
The UV–vis absorption (black) and fluorescent emission spectra of aqueous solution of MIONs-Rh 110 conjugate after extensive dialysis, insert shows optical image in ambient light (**left**), and in UV light (**right**).

MIONs were conjugated with Rh 110 by following the procedure described in Section 2.6. Aqueous solution of MIONs-Rh 110 conjugates, after extensive dialysis for three nights against distilled water, showed fluoresce under UV light as shown in the Figure 11. As a control, a dye solution with same molar concentration was also dialyzed for three nights. It was noted that the control experiment with equivalent concentration of Rh 110 did not exhibit any fluorescence after dialysis, which was the indication of attachment of Rh 110 with MIONs.

## 4. Conclusions

Biocompatible ultra-small magnetic iron oxide (Fe_3_O_4_) NPs were prepared by co-precipitation of aqueous iron precursor solution at high temperature in the presence of water-soluble thioether end-functionalized polymer ligand PTMP-PMAA. The saturation magnetization of the MIONs was further improved by optimizing the polymer ligand concentration compared to our previous work. The increase in magnetization of the MIONs made it possible to manipulate them with external magnets in their aqueous solution, which increases their scope of applications. Furthermore, it was found that these MIONs were fairly uniform in size and could be dried by solvent evaporation, which could be stored for a long time. The dried powders of MIONs were readily re-dispersible in water without any significant change in their size and size distribution. MIONs aqueous dispersion was highly stable, even at high salt concentration and also over a wide range of pH. The concentration of polymer ligand showed significant effect on the size and magnetic properties of MIONs. MIONs were found to be highly biocompatible as determined by MTT-assay. The bio-applicability of MIONs demonstrated by successfully coupling them with fluorescent dye Rhodamine (Rh 110) through formation of amide bond linkage between carboxylic acid (–COOH) groups of MIONs and primary amine (–NH_2_) groups of dye. Similarly, rich surface chemistry of these MIONs stabilized with PTMP-PMAA can be exploited for the simultaneous conjugation of a variety of therapeutic, targeting, and labeling agents for several bio-analytical and drug delivery applications.

## Figures and Tables

**Table 1:** Molecular weight of 2% PTMP-PMAA.

Sample	MAA/PTMP (mol/mol)	Molecular weights (g/mol)			Yield (%)
		*M*n	*M*w	PDI	
2% PTMP-PMAA	100/2	5,850	7,420	1.2	82
